# Intracapsular neck of femur fractures secondary to civilian gunshot injuries: an inter- and intra-observer agreement study on classification and treatment using the AO/OTA classification

**DOI:** 10.1007/s00590-024-04015-4

**Published:** 2024-06-07

**Authors:** Sithombo Maqungo, Andrew Nicol, Maritz Laubscher, Kaylin Williams, Simon Graham, Michelle Henry, Ntambue Kauta, Kirsty Berry

**Affiliations:** 1 Orthopaedic Trauma Service, Division of Orthopaedic Surgery, University of Cape Town, Groote Schuur Hospital, University of Cape Town, H49 Old Main Building, Observatory, Cape Town, 7925 RSA; 2 Trauma Care & Injury Prevention, Division of Global Surgery, University of Cape Town, Cape Town, RSA; 3 Trauma Unit, Division of General Surgery, University of Cape Town, Cape Town, RSA; 4 Liverpool Orthopaedic and Trauma Service, Liverpool University Teaching Hospital Trust, Liverpool, UK; 5 Nuffield Department of Orthopaedics, Rheumatology & Musculoskeletal Sciences, Oxford Trauma and Emergency Care, University of Oxford, Oxford, UK; 6 Liverpool School of Tropical Medicine, Liverpool, UK; 7 Centre for Higher Education Development, University of Cape Town, Cape Town, RSA

**Keywords:** Gunshot, Neck of femur, Treatment options, Classification, Reliability

## Abstract

**Purpose:**

Numerous classification systems have been developed for neck of femur fractures, but none have been tested for reliability in gunshot injuries. Our primary objective was to assess the inter-observer and intra-observer reliability of the AO/OTA classification system when applied to intracapsular neck of femur fractures secondary to low-velocity civilian gunshots wounds (GSWs). Our secondary objective was to test the reliability of the AO/OTA classification system in guiding surgeon treatment choices for these fractures.

**Patients and methods:**

Eighteen reviewers (six orthopaedic traumatologists, six general orthopaedic surgeons and six junior orthopaedic fellows) were given a set of 25 plain radiographs and CT scans of femur neck fractures secondary to GSW. For each clinical case, all reviewers selected a classification as well as treatment option from a list of given options. Inter-observer reliability was measured at the initial classification. The exercise was repeated 10–12 weeks later by the same 18 reviewers to test intra-observer reliability.

**Results:**

The Fleiss kappa values indicate only slight agreement amongst raters, across all experience levels, for both injury classification and treatment. Intra-observer agreement was fair across all experience levels for both injury classification and treatment.

**Conclusion:**

The AO/OTA classification showed only slight reliability in classification of gunshot fractures of the femur neck. With only fair reliability, it also failed to guide surgical treatment thus rendering its routine use in daily clinical practice of questionable value.

## Introduction

Gunshot fractures of the hip joint are relatively rare injuries with notoriously poor outcomes [1, 2]. No reference standard exits for the classification and treatment of these devastating injuries. A number of classification systems have been used for intracapsular fractures of the femur neck, but none have found universal acceptance due to overall poor reliability.

The AO/OTA classification is at present the most comprehensive classification system used [3]. It considers level of the fracture and degree of displacement as well as the angle of the fracture lines. Several studies have however shown it to have poor reliability [4, 5]. The Garden classification and Pauwels’ classification are also widely used, but they also have the shortcoming of poor reliability [6, 7].

Previous neck of femur (NOF) fracture reliability studies have been performed on closed fractures, frequently from low energy falls. No inter-observer and intra-observer reliability studies have been performed on classification and treatment for NOF fractures following penetrating injuries, including civilian gunshot injuries. The rarity and complexity of these injuries, together with the potential for poor outcomes and associated morbidity, necessitate a further quest for evidence-based medicine approach.

### Aims

We therefore set out to:

- Assess the inter- and intra-observer agreement between surgeons in the classification of these injuries in a high-volume clinical setting.
- Analyse its accuracy in guiding the choice of treatment.
- Determine the effect of clinician experience on level of agreement.

## Methods

**Fig. 1:**
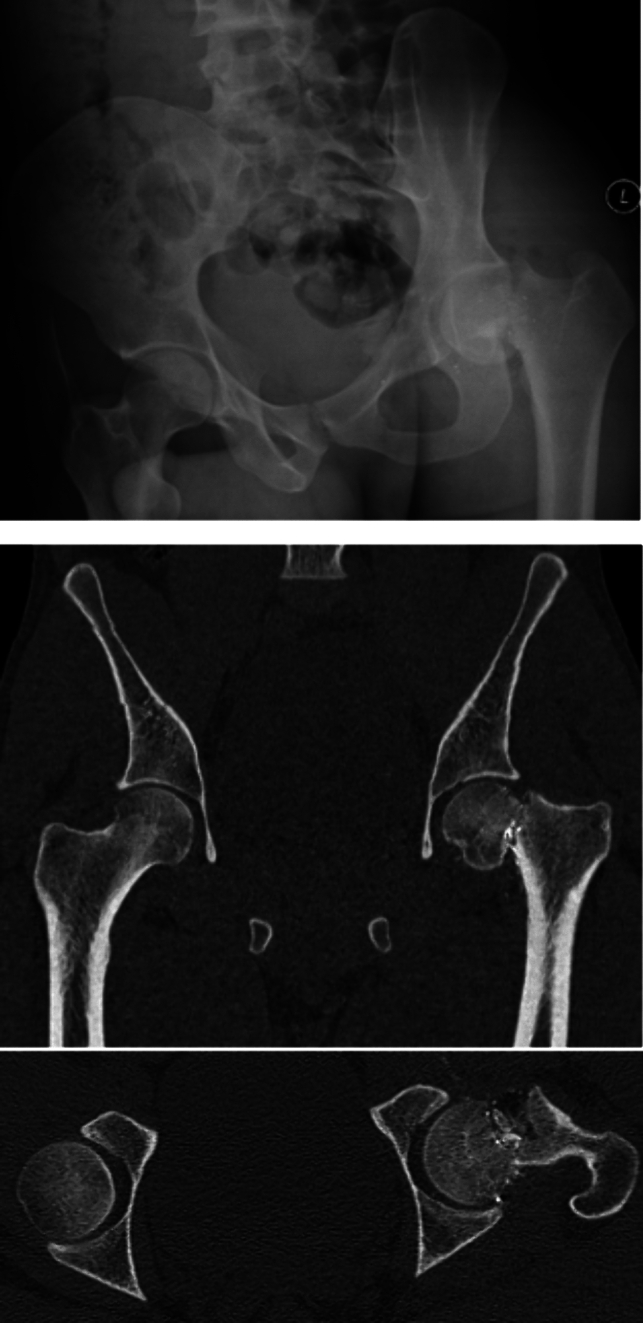
Case example

This observational study was performed using a fixed panel of 18 observers who answered a set of questions regarding classification and treatment by analysing X-rays and CT scans of 25 cases with NOF fractures secondary to civilian gunshot injuries. A case example is shown in Fig. 1. The reviewers included orthopaedic trauma specialists (*n* = 6) and general orthopaedic specialists (*n* = 6) as well as orthopaedic fellows in training (*n* = 6). They were from a total of eight different institutions. Cases were extracted from a single institution’s orthopaedic trauma database between 2016 and 2021.

**Fig. 2:**
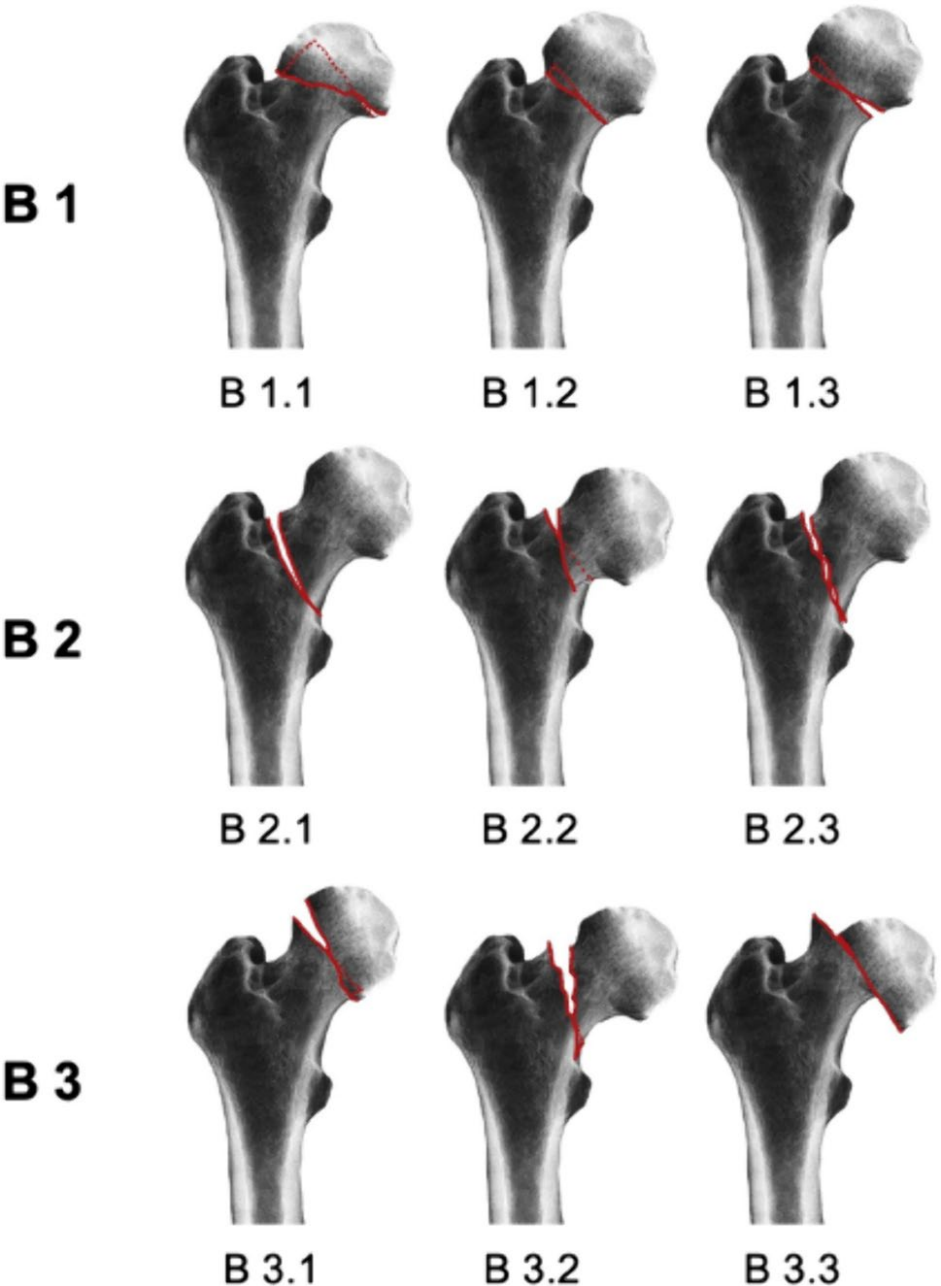
AO/OTA classification

Each reviewer received the AO/OTA fracture classification reference. This consists of nine subtypes in total, based on location of the fracture type (Fig. 2). All the reviewers were blinded to the treatment subsequently received by each patient. For each clinical case, they selected a classification as well as treatment option from a list of given options. There was no time limit imposed in order to allow for accurate assessment.

The interpretation was done over 2 rounds (Time 1 and Time 2), 10–12 weeks apart, without reference to their previous selections. For the second round, the cases were presented in a different order. The first-round classifications and treatment choices were used for inter-observer analysis and the second round for intra-observer analysis.

Study data were collected and managed using REDCap (Research Electronic Data Capture) electronic data capture tools.

## Statistical analysis

Statistical analysis was performed by calculating the Cohen kappa value using SPSS 14.0 statistical software (IBM, Armonk, USA) for intra-observer reliability. In order to calculate the multirater kappa for inter-observer agreement, we used Fleiss kappa values.

We interpreted the kappa value coefficients according to the guidelines proposed by Landis and Koch: less than 0.00 equals poor reliability, 0.00 to 0.20 represents slight reliability, 0.21 to 0.40 fair reliability, 0.41 to 0.60 moderate reliability, 0.61 to 0.80 substantial agreement and 0.81 to 1.00 almost perfect agreement [8].

## Results

The Fleiss kappa values indicate only slight agreement amongst raters, across all experience levels, for both injury classification and treatment (Table 1). Intra-observer agreement was fair across all experience levels for both injury classification and treatment (Table 1).

**Table 1:** Agreement before consolidation of AO OTA categories

Experience level	AO/OTA	Reliability	Treatment	Reliability
*Inter-observer agreement*				
All	0.087	Slight	0.031	Slight
Specialist trauma	0.067	Slight	0.042	Slight
General orthosurgeons	0.047	Slight	0.008	Slight
Fellows	0.110	Slight	0.003	Slight
*Intra-observer agreement*				
All	0.292	Fair	0.383	Fair
Specialist trauma	0.236	Fair	0.331	Fair
General orthosurgeons	0.378	Fair	0.464	Moderate
Fellows	0.262	Fair	0.380	Fair

For the total cohort, the inter-observer agreement for classification was 0.087 representing slight agreement. When broken down to the three subcategories based on experience, trauma surgeons had 0.067, general orthopaedic surgeons had 0.047 and fellows had 0.110 agreement, all representing slight reliability.

For the total cohort, the inter-observer agreement for treatment was 0.031 representing slight reliability. When broken down to the three subcategories, trauma surgeons had 0.042, general orthopaedic surgeons had 0.008 and fellows had 0.003 agreement, all representing slight reliability.

For the total cohort, the intra-observer agreement for classification was 0.292 representing fair reliability. When broken down to the three subcategories, trauma surgeons had 0.236, general orthopaedic surgeons had 0.378 and fellows had 0.262, all representing fair reliability.

For the total cohort, the intra-observer agreement for treatment was 0.383 representing fair reliability. When broken down to the three subcategories, trauma surgeons had 0.331 and fellows had 0.380, all representing fair reliability. With a rating of 0.464, only general orthopaedic surgeons demonstrated moderate reliability.

**Fig. 3:**
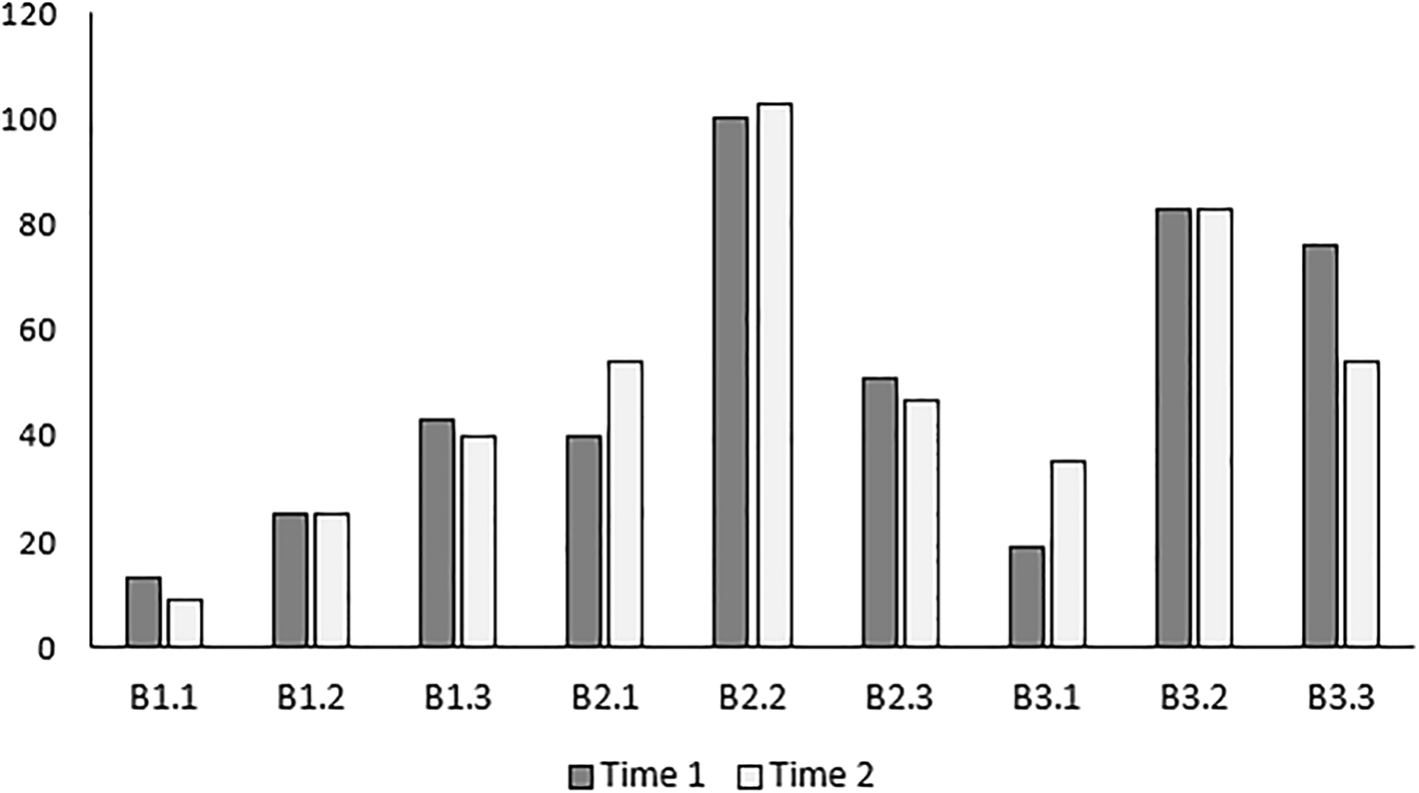
Classification selections

The most common classification types were B2.2 and B3.2 at both rounds of assessment (Time 1 and Time 2) (Fig. 3).

We then consolidated the fracture groups into B1, B2 and B3 without the subclassifications (Table 2). In this exercise, for the total cohort inter-observer agreement for classification, it was 0.146 representing slight reliability, signalling no change when compared to the extended classification. Intra-observer agreement however improved slightly to 0.436 representing moderate reliability.

**Table 2:** Agreement after consolidation of AO OTA categories

Experience level	AO/OTA – 9 categories	Reliability	AO/OTA – 3 categories	Reliability
*Inter-observer agreement classification*				
All	0.087	Slight	0.146	Slight
Specialist trauma	0.067	Slight	0.130	Slight
General orthosurgeons	0.047	Slight	0.130	Slight
Fellows	0.110	Slight	0.140	Slight
*Intra-observer agreement classification*				
All	0.292	Fair	0.436	Moderate
Specialist trauma	0.236	Fair	0.350	Fair
General orthosurgeons	0.378	Fair	0.557	Moderate
Fellows	0.262	Fair	0.402	Fair

**Fig. 4:**
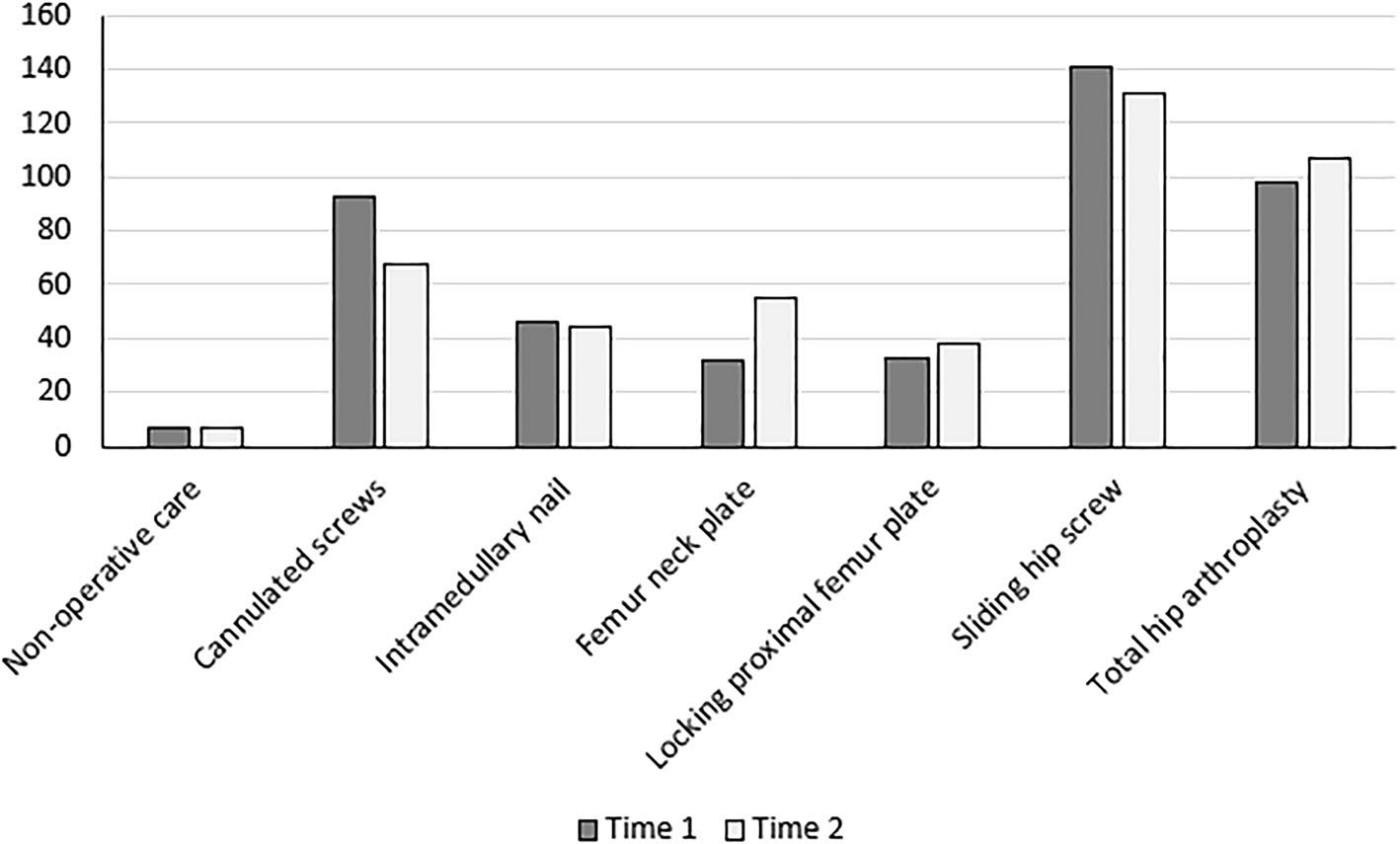
Treatment selections

The three most common implant choices were sliding hip screw (*n* = 141), total hip arthroplasty (*n* = 98) and cannulated hip screws (*n* = 93) at Time 1. At Time 2 observation, the top 3 remained the same but the order changed as follows: sliding hip screw (*N* = 131), total hip arthroplasty (*n* = 107) and cannulated screws (*n* = 68). See Fig. 4.

## Discussion

Gunshot fractures of the hip joint have notoriously poor outcomes, and when treated with internal fixation, they have high complication rates such as non-union, failure of fixation and avascular necrosis [9]. For hip fractures, the anatomical configuration and therefore classification generally determines the treatment option to be adopted. In this study, we assessed the commonly used AO/OTA classification for its inter- and intra-observer reliability in classifying gunshot fractures of the femur neck. We also assessed it for its reliability in guiding treatment choices. This is the first study to our knowledge to report on reliability of this classification in NOF fractures secondary to civilian gunshots. We have found only slight reliability amongst all experience levels when it comes to classification and fair reliability in guiding treatment options.

Ideally, a fracture classification system should have good inter-observer and intra-observer reliability and should also be able to provide information on stability, guide treatment interventions and allow for scientific comparisons of ‘like with like’. It should also be able to predict anatomic and functional outcomes and be appropriate for daily clinical practice and audit [10, 11]. Femur neck fractures secondary to firearm injuries differ when compared to closed (commonly fragility) fractures due to the higher energy imparted and the inherent comminution that is present in all fractures.

Various classification systems have been proposed to classify intracapsular hip fractures, but none have found universal acceptance. The most commonly used system is that of Garden who divided them into four groups based on impaction or degree of displacement on anteroposterior radiographs [12]. Many subsequent studies however have doubted the value of the Garden system due to its poor reliability [4, 6, 13–18]. Parker was the first to show that the difference in the rates of fracture healing between Garden types III and IV was not sufficient to justify separating these two grades [14].

The Pauwel classification has also been used commonly. It has three subtypes, and it considers the angle of the fracture line relative to the femur shaft. It associated a greater vertical shear fracture line with an increase in incidence of non-union and malunion. It too however has been shown to have poor inter-observer reliability and has also been shown to be not predictive of non-union or avascular necrosis [7, 19]. Pauwel classification is also fraught with difficulties with accurate measuring of the fracture line angle due to rotation of the femur [20]. As these are penetrating injuries, often affecting younger patients compared to blunt trauma, applying the available classification systems has been challenging in the clinical setting.

The AO/OTA classification has also been found to not be reliable in both closed intracapsular and extracapsular fractures of the femur neck [21, 22]. In this study, we have reached similar findings and a similar conclusion that it is too complicated for routine clinical use. Even when we collapse the subcategories and group together B1, B2 and B3 fractures without the subdivisions, the results remain the same, slight reliability, even though there was minor improvement, it was negligible to affect the rating. In previous studies, there has been an improvement in agreement rating when the AO classification was simplified into fewer categories [21]. This has not been the case in our study.

Reproducible and accurate fracture classification is important to guide the surgical implant of choice as well as the prognosis of the injury in terms of malunion, non-union and avascular necrosis. When one takes into account experience levels amongst the observers, only general orthopaedic surgeons could reach fair agreement on treatment, with many opting for a sliding hip screw device (Fig. 4). Prior to our current study, no agreement studies have been performed on treatment choices for these injuries. And it is clear from this data that the low reliability meant treatment choices were also unreliable as many surgeons changed their opinion of treatment choice during the second round.

The high proportion of total hip arthroplasty as a treatment choice was unexpected given the average age of 28 years for the cohort. There is no strong evidence to support this practice. Only sporadic case reports have reported on arthroplasty being performed much later in a staged manner, rather than in the acute setting [23–25].

## Limitations

The low numbers are a recognised limitation of our study, but these are relatively rare injuries collected over an extended period. Our unit is a high-volume Level 1 Trauma Centre in an urban area with a high burden of gunshot injuries. All observers practised in the same country, albeit at different institutions, so the results may not be generalisable to other countries or regions.

## Conclusion

We have found the AO/OTA classification to have only slight intra- and inter-observer reliability in classifying intracapsular civilian gunshot fractures of the femoral neck. The experience level of the reviewers did not improve its reliability. With only fair reliability, it also failed to guide surgical treatment thus rendering its routine use in daily clinical practice of questionable value.

Future research needs to focus on developing a reliable classification system for these injuries that is able to both guide treatment and to predict the outcome.17].

## Declarations

### Human and animal participants

The study does not involve the use of animals by any of the authors.
